# Digits-In-Noise Hearing Test Using Text-to-Speech and Automatic Speech Recognition: Proof-of-Concept Study

**DOI:** 10.1177/23312165251367625

**Published:** 2025-10-01

**Authors:** Mohsen Fatehifar, Kevin J. Munro, Michael A. Stone, David Wong, Tim Cootes, Josef Schlittenlacher

**Affiliations:** 1 Manchester Centre for Audiology and Deafness (ManCAD), 5292 University of Manchester, Manchester, UK; 2 Manchester Academic Health Science Centre, Manchester University Hospitals NHS Foundation Trust, Manchester, UK; 3 Leeds Institute of Health Sciences, 70535 University of Leeds, Leeds, UK; 4 Centre for Imaging Sciences, 5292 University of Manchester, Manchester, UK; 5 Department of Speech, Hearing and Phonetic Sciences, 4919 University College London, London, UK

**Keywords:** digits-in-noise test, automated test, automatic speech recognition, text-to-speech, speech-in-noise test

## Abstract

This proof-of-concept study evaluated the implementation of a digits-in-noise test we call the ‘AI-powered test’ that used text-to-speech (TTS) and automatic speech recognition (ASR). Two other digits-in-noise tests formed the baselines for comparison: the ‘keyboard-based test’ which used the same configurations as the AI-powered test, and the ‘independent test’, a third-party-sourced test not modified by us. The validity of the AI-powered test was evaluated by measuring its difference from the independent test and comparing it with the baseline, which was the difference between the Keyboard-based test and the Independent test. The reliability of the AI-powered test was measured by comparing the similarity of two runs of this test and the Independent test. The study involved 31 participants: 10 with hearing loss and 21 with normal-hearing. Achieved mean bias and limits-of-agreement showed that the agreement between the AI-powered test and the independent test (−1.3 ± 4.9 dB) was similar to the agreement between the keyboard-based test and the Independent test (−0.2 ± 4.4 dB), indicating that the addition of TTS and ASR did not have a negative impact. The AI-powered test had a reliability of −1.0 ± 5.7 dB, which was poorer than the baseline reliability (−0.4 ± 3.8 dB), but this was improved to −0.9 ± 3.8 dB when outliers were removed, showing that low-error ASR (as shown with the Whisper model) makes the test as reliable as independent tests. These findings suggest that a digits-in-noise test using synthetic stimuli and automatic speech recognition is a viable alternative to traditional tests and could have real-world applications.

## Introduction

Hearing tests are usually undertaken in hospital and clinic environments with specialised equipment and professionally qualified staff. However, these are not always available (Fagan & Jacobs, 2009; Planey, 2019). Additionally, people are slow to seek help when experiencing hearing difficulties and there is an estimated delay of 8.9 years between the time hearing aids are needed to the time of their adoption (Simpson et al., 2019). To solve these problems, there have been attempts to develop remote hearing tests (Almufarrij et al., 2023; Fatehifar, Schlittenlacher, et al., 2024) and there is significant potential for innovation in self-administered hearing tests; specifically, there has been considerable interest in incorporating machine learning into the test procedure to make it less dependent on human supervisors (Fontan et al., 2020; Gonçalves Braz et al., 2022).

Speech audiometry is a common procedure used to evaluate hearing difficulty (Boothroyd, 1968) and can provide early identification of hearing problems. It is often performed by measuring the participant's ability to understand speech presented against a background noise, a so-called speech-in-noise (SIN) test. This approach is more robust in uncontrolled environments compared to pure-tone audiometry (PTA), as it is less reliant on the low background noise obtained in a sound-treated setting. Furthermore, SIN tests are more ecologically valid than conventional PTA since understanding speech in background noise is the primary complaint of hearing-impaired listeners (Healy & Yoho, 2016; Moore et al., 2014; Plomp, 1978).

There are three categories of speech material that are commonly used in SIN tests: (1) sentences, (2) words, of which digits are a specific example, and (3) nonsense syllables.

One of the challenges of SIN tests is creating the test stimuli, as this process is often time-consuming and costly. To overcome this, researchers have suggested using text-to-speech (TTS) which is a technology used to generate synthetic speech stimuli using computers. Kosai et al. (1990) used TTS to synthesise vowels, Polspoel et al. (2025) used it to synthesise digits, and other studies used TTS to generate German words for a SIN test (Ibelings et al., 2022; Nuesse et al., 2019; Ooster et al., 2020).

A further challenge in SIN tests is the need for human supervision to adjust stimulus or noise levels based on whether the participant gave the correct response or not. In these tests, the supervisor sets the noise or stimulus level of the next stimulus to alter the degree of difficulty. The measure of performance is given by a value called the signal-to-noise ratio (SNR); this value, in decibels, defines the relative level of the target signal compared to the interfering noise in the composite stimulus. The test aims to measure the speech reception threshold (SRT) of the person, which is the lowest SNR at which the participant can understand a fixed proportion of the presented stimuli, typically 50%. Negative SNRs are common in SIN tests, which implies that speech can be understood when its (long-term) level is below that of the background noise. Unmasking of the speech from the noise comes from its property as being a temporally varying signal; in short time windows, it can exceed the level of the noise, even at negative SNRs.

An additional challenge in SIN tests is capturing participant responses. Researchers have explored self-administered SIN tests that use various methods to obtain this participant's response (e.g., clicking buttons on the screen or via a telephone keypad). Such methods have been applied to the DIN test (Denys et al., 2018; Folmer et al., 2017; Smits et al., 2004) and simple versions of the SIN test in which participants needed to report only one word (Hisagi et al., 2022; Molander et al., 2013). However, these methods add a layer of complexity to the test, as participants need to operate another device (e.g., a computer) properly to perform the test. This makes the test harder for people who are uncomfortable with, or unable to use, keyboards or touchscreens.

Because of these limitations in response collection, some researchers have employed automatic speech recognition (ASR), a technology that converts spoken language into text, to transcribe the participants’ verbal responses. Researchers have focused mainly on sentence-in-noise tests with ASR transcribing participant responses instead of a clinician (Bruns et al., 2022; Meyer et al., 2015; Nisar et al., 2019; Ooster et al., 2018, 2023). Ooster et al. (2020) created a system with both TTS and ASR that did not need human recorded stimuli or human supervision. However, research on using ASR to get responses in the DIN tests is limited, and only one study has used ASR for transcribing digits in a DIN test (Araiza-Illan et al., 2024).

In previous studies that employed TTS or ASR, the use of digits as stimuli has been relatively uncommon, with only two recent studies identified. Polspoel et al., (2025), synthesised digits using a TTS, trained and provided by Google LLC, aiming to reduce the cost and time needed to create new stimuli. They tested the synthetic stimuli on 48 native Dutch speakers, including both normal-hearing and hearing-impaired, for both English and Dutch digits. Their findings showed that TTS could be successfully used for generating DIN stimuli.

Araiza-Illan et al., (2024) used a pre-trained ASR system to transcribe the Dutch version of the DIN test. They evaluated the impact of ASR errors on SRT measurements by conducting a simulation using the bootstrapping method on DIN results from six native Dutch-speaking adults with normal-hearing. They found that, for up to four triplets with ASR errors per run, the result in SRT variation was within an acceptable range of 0.70 dB.

The current proof-of-concept study combines ideas from both of these studies and uses both TTS and ASR to create a fully self-supervised English-language DIN test with the ability to easily generate the stimuli. A systematic evaluation of a DIN test with these technologies is necessary because the effects of adding TTS and ASR may be quantified more precisely in this simple version of a SIN test before employing them in tests using more complex test material.

For this study, a program was developed to perform the DIN test in two different versions that used different forms of stimuli generation (human generated or synthetically generated from text) and response capturing methods (keyboard or ASR). Apart from the use of TTS and ASR, the two versions shared the same test parameters and source code to quantify the effect of the combined use of the two technologies on test validity and reliability.

In addition to two DIN tests using our developed software, a third DIN test created by another research team was employed to evaluate how closely the results of our two implementations of the DIN test, with differing non-standardized test parameters and no AI involvement, aligned. This, in turn, allowed us to form the baseline and assess the validity and reliability of our developed test.

This proof-of-concept study was designed to evaluate the effect of using TTS and ASR in performing a DIN test, and answer our hypothesis, that an AI (TTS and ASR) based DIN test can be as reliable and valid as a conventional DIN test.

## Method

### Participants

31 participants were recruited based on Daniel's (2012) recommendation for the sample size of a proof-of-concept study.

Potential participants were identified via posters on the university campus, and announcements through the University's website and social media. Additionally, we sent an email to persons who have joined the Manchester Centre for Audiology and Deafness volunteer dataset, some of whom will have hearing loss. This was a convenience sample in which participants were recruited based on their accessibility and willingness to participate.

As this was a proof-of-concept study and the stimuli were limited only to digits, there was no restriction on participants’ native language and nationality, and each participant was compensated £20 in cash for their participation.

To determine whether a participant was to be grouped as either “normal-hearing” or “hearing-impaired”, PTA was performed at 250, 500, 1000, 2000, 4000 and 8000 Hz, separately in each ear, then, following convention, averaged for 500, 1000, 2000 and 4000 Hz in each ear (Haggard et al., 1981). Normal-hearing was defined as an average threshold of ≤ 20 dB HL in the better ear. The upper limit for hearing loss was set at 55-dB HL in the better ear to ensure that the participants could hear the presented number and finish the test without the use of a hearing aid.

### Equipment

PTA test was performed with a calibrated GSI Pello audiometer. To deliver the digit stimuli, a calibrated Scarlett Solo 4th Gen audio interface and Sennheiser HD 650 headphones were used. The built-in microphone of a MacBook Pro M1 was used for recording the response. All the testing was done in a standard sound-treated booth.

### Procedure

This study was pre-registered at the open science framework database (Fatehifar, Munro, et al., 2024) and used STARD reporting guidelines (Bossuyt et al., 2015). This study compares three different triplet DIN (Smits et al., 2013) methods. In all three tests, participants were presented with three random, non-repeating digits mixed with a randomly selected segment of the same 5-min babble noise, delivered to both ears (Shehabi et al., 2025). The three tests were:

1. AI-powered test: This is the main developed test that used TTS to generate the stimulus and ASR to capture participants’ responses.
2. Keyboard-based test: This test was implemented using exactly the same logic, parameters and software as the AI-powered tests. The only difference is that it used the keyboard for participants’ responses and human generated voice stimuli. The purpose of this test was to show the impact of TTS and ASR when everything else was held constant.
3. Independent test: This test was implemented by a separate group of researchers (Shehabi et al., 2025) and used the same human generated speech stimuli as the Keyboard-based test. This test was used to define an independent baseline for accepted levels of reliability and validity.

**Figure 1.**
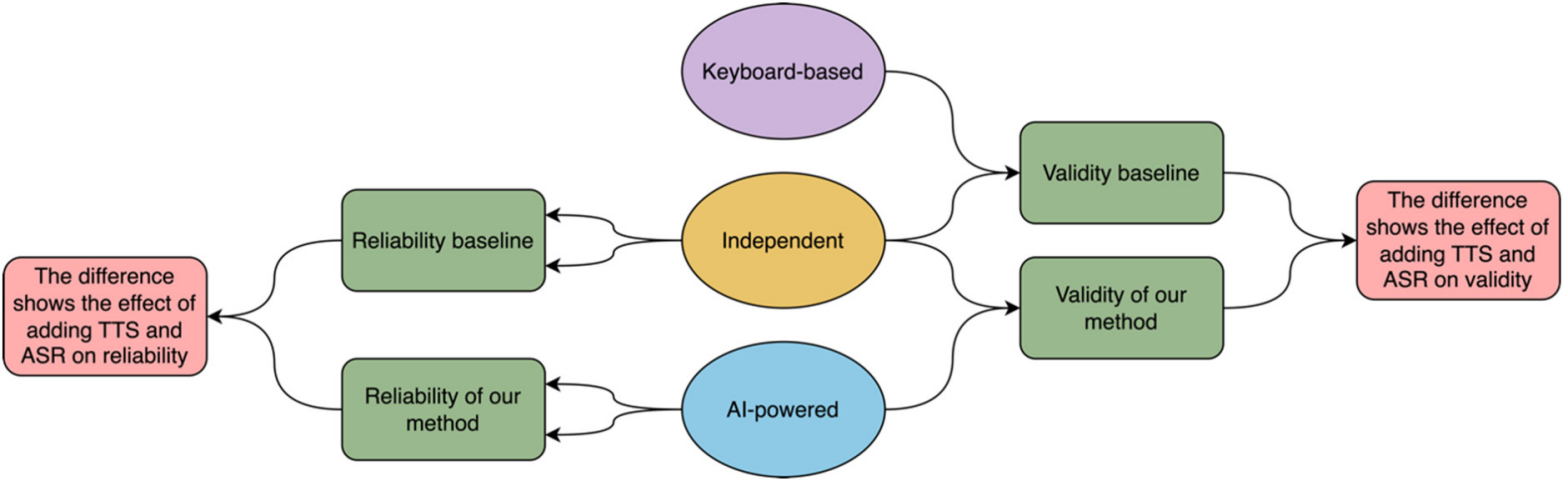
Shows How the Three Tests Were Compared. Each Merged Line Shows a Comparison of Results.

The three implementations of DIN tests that were performed in this study are described in detail in the following section. The list of the configurations of those tests is provided in Table 1. Figure 1 shows how each test was related and compared with other tests.

**Table 1.** Configurations of the Three Involved DIN Tests.

	Independent	Keyboard-based	AI-powered
**Stimuli**	0 to 9	0 to 9	0 to 9
**Noise**	Multi-talker babble noise randomly sampled from a larger file.	Multi-talker babble noise randomly sampled from a larger file.	Multi-talker babble noise randomly sampled from a larger file.
**Presentation level**	Noise and digits level adapted for constant overall level	Digits fixed at 65 dB. Noise was adaptive	Digits fixed at 65 dB. Noise was adaptive
**Speech generation**	Human recorded (Female)	Human recorded (Female)	TTS generated (Female)
**Response Capture**	Select with mouse on screen	Keyboard entry	Spoken repetition
**Correct criteria**	Repeat 3 digits	Repeat 3 digits	Repeat 3 digits
**Stepping rule**	2-Down, 1-Up	2-Down, 1-Up	2-Down, 1-Up
**Step size (dB)**	6 in first phase; 2 in second phase	5 in first phase; 3 in second phase; 1 in third phase	5 in first phase; 3 in second phase; 1 in third phase
**Reversal count**	First phase: 4; Second phase: 6	First phase: 2; Second phase: 2; Third phase: 6	First phase: 2; Second phase: 2; Third phase: 6

#### Tests

##### AI-Powered DIN

This is the main DIN test that was implemented for this proof-of-concept study using TTS and ASR. This test used a TTS model to synthesise the stimuli during the session and an ASR model to automatically transcribe participants’ responses. The transcription was then evaluated by the computer to determine the correctness of the answer and the required adjustment of the SNR.

This test presented digits in the English language at a root mean square (RMS) sound pressure level (SPL) of 65 dB and used speech-babble noise with a sample rate of 22.05 kHz and bandwidth of 10.4 kHz (Ewing Foundation, n.d.). Initially, the speech-babble noise level was set to 60 dB SPL (equating to an SNR of +5 dB) and then adjusted based on participants’ responses, with an upper limit of 80 dB SPL. The noise started 0.5 s before the first digit and continued for 0.5 s after the end of the last digit.

After presenting the stimuli, participants were asked to repeat the numbers they heard aloud. The participant had to repeat all three digits in the same order as they were presented for them to be scored correct (e.g., a response of 1-6-2 is incorrect if the stimuli were presented in the order 6-1-2). Based on the participant's response, the SNR for the next stimulus was altered by changing only the noise level. The SNR decreased (by increasing the noise level) after two correct responses and increased (by decreasing the noise level) after one incorrect answer (Levitt, 1971).

**Figure 2.**
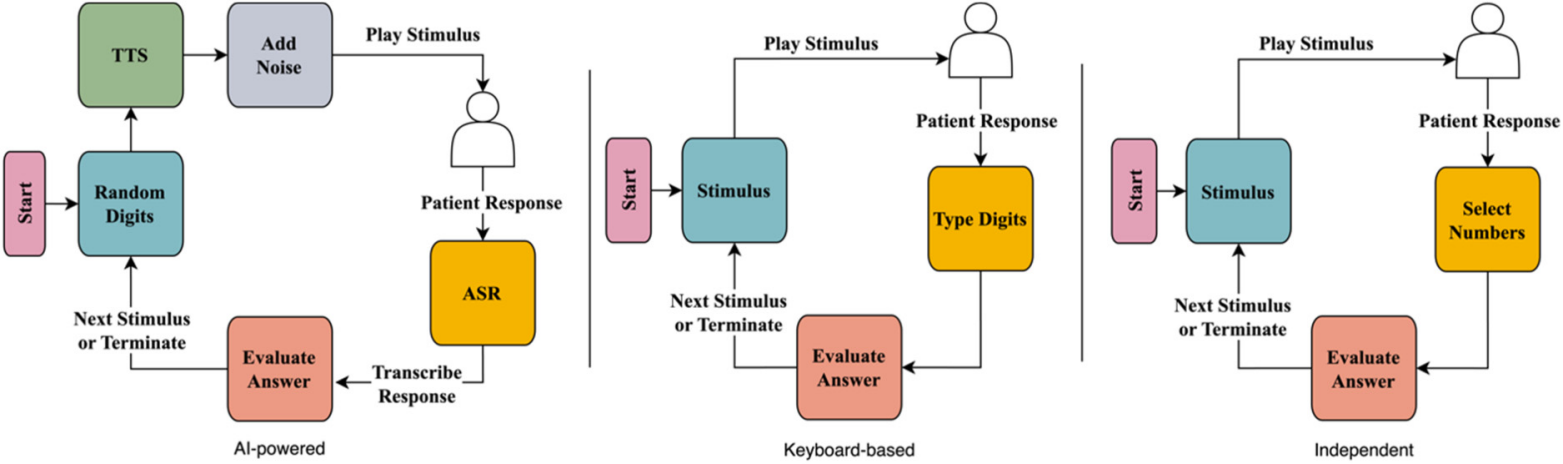
Comparison of Procedures for Each of the Three DIN Tests.

We define a *reversal* as a change in the direction of SNR from increasing to decreasing, or vice versa. The noise level adjustment was set to a step size of 5 dB for the first two reversals (phase 1), 3 dB for the next two reversals (phase 2) and 1 dB for the last six reversals (phase 3). This process continued until there had been ten reversals. The SRT was determined by averaging the SNRs in phase 3. These choices gave good results in other psychoacoustic tests (Schlittenlacher et al., 2020, 2022). If there were six correct responses at an SNR of −15 dB, the results were recorded as ‘−15 dB or lower’. The procedure of this test is shown in Figure 2.

Participants’ vocal recordings and the ASR transcription were saved for future analysis and for measuring the performance of the ASR system.

*Automatic Speech Recognition. *The ASR system used for transcription was an end-to-end, off-the-shelf, pre-trained ASR from SpeechBrain (Ravanelli et al., 2021). This general-purpose ASR, trained on LibriSpeech (Panayotov et al., 2015), can transcribe full sentences and not just numbers. The trained model is freely accessible via HuggingFace^1^.

As this was a general-purpose ASR, its output was not limited to only numbers, and it could make mistakes for digits that are similar to some other words (e.g., homophones such as ‘eight’ and ‘ate’). To address this issue, the first author created a dictionary (available in the supplementary materials) of these types of digit and word confusions with 62 items based on the piloting of the study. This dictionary was used to translate the response to the corresponding digit. The advantage of general-purpose ASR software, rather than one optimised for digits, is that it may generalise to future work on more complex SIN tests.

*Text-To-Speech. *The TTS system used a pre-trained model^2^ from SpeechBrain that generated speech with a sampling frequency of 22.50 kHz. The TTS used Tacotron2 (Shen et al., 2018) trained on LJSpeech (Keith Ito & Linda Johnson, 2017) to produce Mel spectrograms from the input text and HiFi-GAN (Kong et al., 2020) as a vocoder to convert the Mel spectrograms of a randomly generated three-digit number to waveforms of speech signals.

TTS was used to generate synthetic speech during the session, which was not strictly necessary for this study, as the stimuli could have been pre-generated. However, since this was a proof-of-concept study, it was decided to run the TTS during the session without adjusting the TTS output. Generating the stimuli in real-time allowed us to assess the effectiveness of the TTS system and determine if it could be used for future SIN tests that require real-time stimuli generation (e.g., a more conversational SIN test or a SIN test with the topic adaptable to the interests of a child).

##### Keyboard-Based DIN

The main differences between the Keyboard-based test and the AI-powered test were (1) digits were recorded by a human speaker and not synthesised during the test and (2) participants were required to type their response with the keyboard instead of verbally repeating the presented numbers. In this test, human recorded digits from Moore et al., (2019) were used.

##### Independent DIN

To measure what could be an acceptable difference between two different DIN tests, an independently implemented DIN test (Shehabi et al., 2025) was used with default configurations and rules that were not altered by the authors.

There was no established reference DIN test, so selecting this particular implementation was an arbitrary decision, made primarily because we had easy access to the test and the researcher who implemented it.

This test used the same speech and noise samples as for the Keyboard-based test, and the RMS level of the overall stimulus (digits mixed with noise) was held constant during the session. The initial SNR was +2 dB, and the signal level was set to 65 dB SPL at the beginning of the test.

Based on the participant's response, the SNR for the next stimulus was altered by changing both the noise and signal levels (the digits). Both noise and the digit levels were adjustable to ensure that stimuli were always audible and not too loud. For example, if the noise level was already high, increasing it further to achieve a lower SNR might make the noise uncomfortable for the participant. Therefore, instead of increasing the noise, the digit volume was reduced to achieve the desired SNR.

After the stimuli were presented, the participant was asked to select with a mouse the digits they heard by clicking on a computer screen. Similar to the other two tests, for a response to be considered correct, the participant had to enter all three digits in the presented order. The SNR level adjustment was set to a step size of 6 dB for the first four reversals (phase 1) and 2 dB for the next six reversals (phase 2). The procedure of this test is provided in Figure 2.

There were two main differences between the configuration of this test and the two developed tests:

1. The step sizes. The Independent test had two phases with 6- and 2-dB step sizes, while the two developed tests had three phases with 5-, 3- and 1-dB step sizes.
2. The method of adjusting the noise and digits signal. The Independent test changed both the digits and noise level while the Keyboard-based kept a fixed level of 65 dB SPL for digits and only changed the noise level.

A diagnosis based on the results of a DIN test should not depend on these choices, but there is no standard for these parameters. Thus, the Independent test was deliberately chosen in a way to have these differences and not be the same as our implementation. This enabled us to compare our implementation to a previously tested and published test while still being able to determine separately the effect of ASR and TTS by comparing the AI-powered test with the Keyboard-based test.

#### Test Session

**Figure 3.**
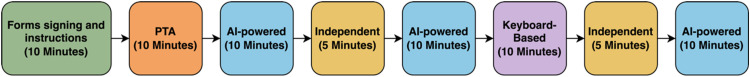
Steps in the Testing Session.

Participants were invited for a single 90-min session in a sound-treated hearing booth. The tests and the order of performing these are shown in Figure 3.

The session started by obtaining consent and was followed by PTA to determine the participant's hearing level. After ensuring that the participant met the inclusion criteria, their SRT was measured six times; 3 times with the AI-powered test (blue boxes), twice with the Independent test (yellow boxes) and once with the keyboard-based test (purple box).

The AI-powered test and the Independent test were performed multiple times during the session to measure their (short-term) test–retest reliability. The keyboard-based test was done only once because we were mainly interested in comparing its validity (the differences with the Independent test) with the validity of the AI-powered DIN.

The test order was fixed since there is little learning effect in general for the DIN test (Smits et al., 2013). Thus, it was expected that the order of conducting the test would not significantly affect the measurement of SRT.

### Analysis

#### Performance of the ASR System

Participants’ responses were transcribed from audio recordings by the author after the session, and this was used for evaluating the performance of ASR in each run of the test. Performance was defined using equation (1). The numerator is the number of mistakes that happened because of ASR's incorrect transcription and the denominator is the number of incorrect responses (both from ASR mistakes and participants not discriminating the stimuli).

When a response was wrong due to mistakes by both the participant and the ASR, it was not counted toward the ASR mistakes as it would not have affected the calculation of the next SNR. Additionally, it is very unlikely for a wrong answer to be mistakenly transcribed to a correct one and did not happen during the testing, thus it was not included in this report. Note that this definition of an ASR error rate does not give the error as the proportion of all data but as a proportion of all errors that were made in the test. Thus, it does not consider correct trials and yields higher values than metrics like the word error rate.

(1) $$\mathrm{ASR}\;\mathrm{error}\;\mathrm{rate}=\frac{ASRMistakes}{AllMistakes}*100\text{\%}$$

Additionally, the recorded speech was transcribed using the Whisper model (Radford et al., 2022), a highly accurate ASR system that could not be used in real time due to its high computational demands and resulting delays. The error rates of the two ASR systems were compared to evaluate how future improvements in ASR technology might influence the performance of the test.

#### Performance of the TTS System

To assess the performance of the TTS system, the Short-Time Objective Intelligibility (STOI) metric (Taal et al., 2010) was measured for 30 randomly selected TTS-generated stimuli and compared to the STOI of natural recordings across SNRs ranging from −15 to 15 dB with a step size of 1 dB. This metric assesses the intelligibility of speech at varying noise levels, providing a score between 0 and 1, where a score of 1 indicates the highest intelligibility.

Additionally, the naturalness of the same 30 TTS samples was evaluated using the NISQA-TTS tool (Mittag & Möller, 2020), and their Mean Opinion Score (MOS) was reported. The MOS is a subjective rating of naturalness, where stimuli are scored on a scale from 1 to 5, with 5 indicating the most natural.

#### DIN Methods Comparisons

To compare different DIN methods, Bland-Altman (B-A) analyses and Pearson correlations were reported. The test-retest reliability of each test (i.e., how close the results of a test are if they are done multiple times for the same person) was analysed using the same methods. For the B-A plot, the mean of the difference and the Limits of Agreement (LoA) were reported. The Pearson correlation coefficients and the root-mean-square errors (RMSEs) were reported to enable comparisons to the literature.

#### Defining the Baselines

B-A analysis was used as it is widely adopted in health sciences for comparing different measurement methods (Giavarina, 2015). However, this test is rarely used in studies reporting test–retest reliability and validity of different DIN tests. Thus, the Independent test and the keyboard-based test were used to define these baselines.

For validity, the baseline is the difference between the Independent test and the keyboard-based test. This shows how much difference is expected between two different implementations of the DIN that also capture responses differently (mouse versus keyboard). The overall validity of the AI-powered test can be obtained by comparing it to the established Independent test. The effect of adding TTS and ASR alone on validity can be obtained by comparing the difference between the AI-powered test and the Keyboard-based test. How the tests are compared is shown in Figure 1.

For reliability, the test-retest reliability of the Independent test was used as the baseline. It could be expected that this would result in the smallest LoA since this was a validated and published test. If the AI-powered test achieves a similar LoA it means that the test is reliable. How the tests are compared is shown in Figure 1.

#### Indices

As mentioned in the previous section, the AI-powered DIN and the Independent test were run multiple times. To compare different methods of performing the DIN test, the results of the different runs for each method need to be aggregated into one.

For estimating validity, the average of all the runs of the Independent test and the AI-powered test was used to measure a better estimate of the SRT. For estimating the test-retest reliability, the first two runs of the Independent test and the AI-powered test were used.

#### Removing Outliers

In general, ASR of speech-in-quiet can achieve error rates lower than 2% (Zhang et al., 2022). However, obtaining similarly low error rates for accented speech is an active research field (Prinos et al., 2024; Zhang et al., 2022) that is not the purpose of the present study.

In the present study, there was no limitation on participants’ first language, thus, for some participants who spoke with a strong accent, the ASR might perform poorly due to mis-transcription. To mitigate this and show the potential of the AI-powered DIN test, participants with an ASR error rate exceeding 40% were removed from the results. Note that this percentage represents the proportion of errors caused by the ASR system out of all the mistakes, not out of all the responses. This threshold was chosen based on an analysis of the collected data, aiming to balance retaining as much data as possible while removing participants with a high error rate. This cut-off is high, and systems that achieve better error rates are likely to be available for most speakers. When comparing the validity, this threshold was applied to the ASR error rate averaged across the three runs. When measuring the reliability, this threshold was applied to the first and second runs of the AI-powered test. If participants had an error rate of more than 40% in one of the two first runs, they were excluded.

Additionally, 40% align with the findings of Araiza-Illan et al. (2024), which suggest that four triplets with errors from the ASR system do not impact SRT measurement. Based on the collected data, our participants made an average of 10 to 11 mistakes per round, making 40% equivalent to approximately four mistakes.

When comparing the Keyboard-based test with the Independent test, one hearing-impaired participant showed an atypically high difference on these tests. This comparison was removed from further analysis since it would have dominated the reported single-value measures.

### Deviation from the Registered Protocol

There were four deviations from the registered protocol:

1. We included a second hypothesis to verify whether the software was functioning correctly before integrating TTS and ASR into the system. This hypothesis was not considered to focus on the primary research question and assess the impact of TTS and ASR on SRT measurement. This deviation did not affect the testing process and only influenced how the results were presented.
2. Another deviation involved how the results of the AI-powered and independent tests were combined to assess validity. Initially, we planned to use the first run of the independent test and the run with the lowest ASR error for the AI-powered test. Instead, we opted to average the runs to provide a more representative measure of the actual value, rather than selecting the best outcome.
3. We also revised the approach to evaluating validity. The registered protocol proposed comparing the alignment of the AI-powered test with the Independent test to the reliability of the independent test. However, this comparison proved ineffective since the two tests measured different aspects using different methods. Instead, we established a baseline for the expected difference between two implementations of the DIN test (AI-powered and Keyboard-based) and compared this difference to the difference between the AI-powered and Independent tests. This allowed us to isolate the impact of TTS and ASR while keeping all other factors constant.
4. We did not intend to remove any data when comparing the Keyboard-based test and the Independent test; however, after collecting the data, we decided to exclude a participant with large discrepancies between the two tests to avoid setting an exaggerated and higher LoA as the baseline.

## Results

For this study, 31 participants were recruited. The 10 hearing-impaired participants had an average PTA threshold of 36 ± 11 dB HL in their better ear, while the 21 with normal-hearing had an average PTA threshold of 3 ± 5 dB HL in their better ear.

Tables 2 and 3 provide a summary of the results, with and without outliers removed. In comparisons involving the AI-powered test, five participants were excluded during validity assessment and seven during reliability assessment. This was due to the ASR system producing high error rates (>40%). For the comparison between the independent test and the keyboard-based test, one outlier that dominated the LoA was removed. In both tables, the baseline values are highlighted using bold font.

**Table 2.** Summary of B-A Results for all Three Tests, Showing Mean ± 95% Limits of Agreement. The Bold Fonts Show the Baselines. Results After Removing Outliers Are Shown in Parentheses.

	Independent	AI-powered
Independent	**−0.4** **±** **3.8 dB**	Same as AI-powered vs Independent
AI-powered	−1.3 ± 4.9 dB (0.6 ± 3.9 dB)	−1.0 ± 5.7 dB (−0.9 ± 3.8 dB)
Keyboard-based	**−0.5** **±** **6.0 dB ** **(−0.2** **±** **4.4** **dB)**	−1.8 ± 5.3 dB (−1.2 ± 4.4 dB)

**Table 3.** Summary of Correlation Results for all Three Tests. Each Cell Shows the Pearson Correlation Coefficient (R) and Root Mean Square Error (RMSE) in dB. The Bold Fonts Show the Baselines.

	Independent	AI-Powered
Independent	***R: 0.92, RMSE: 2.0***	Same as AI-powered vs Independent
AI-Powered	R: 0.84, RMSE: 2.8 (R: 0.91, RMSE: 2.0)	R: 0.81, RMSE: 3.0 (R: 0.92, RMSE: 2.1)
Keyboard-based	**R: 0.75, RMSE: 3.0 ** (R: 0.82, RMSE: 2.2)	R: 0.80, RMSE: 3.2 (R: 0.87, RMSE: 2.5)

### Baselines

**Figure 4.**
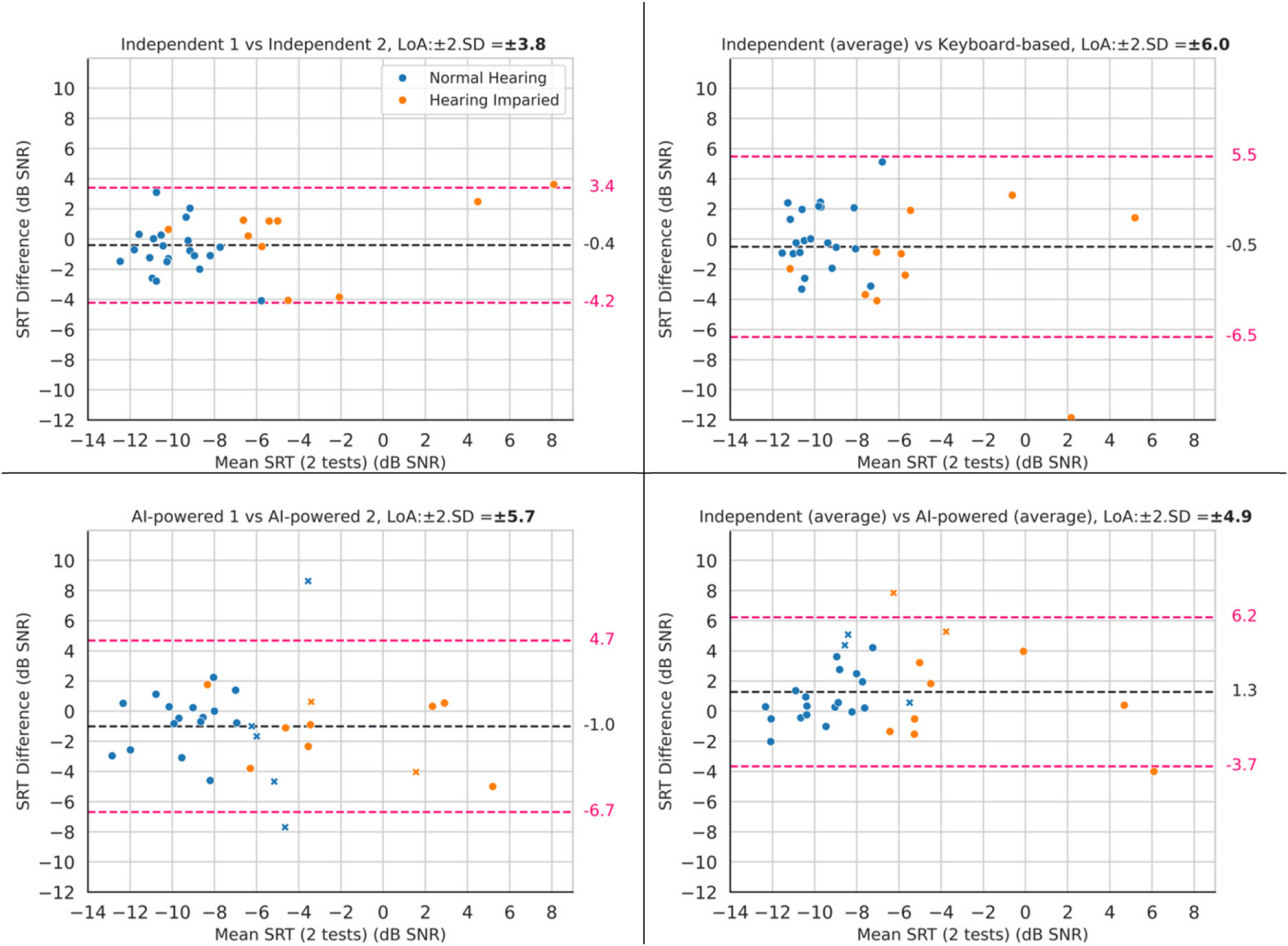
Comparison of B-A plot of the Different Methods of DIN Test. The Blue Marker Represent Participants with Normal-Hearing and the Orange Marker Represent Participants with Hearing Loss. The Markers Shown as Cross are the Outliers with High ASR Error Rate. In this Plot, LoA Stands for Limits-Of-Agreement and SD Stands for the Standard Deviation.

**Figure 5.**
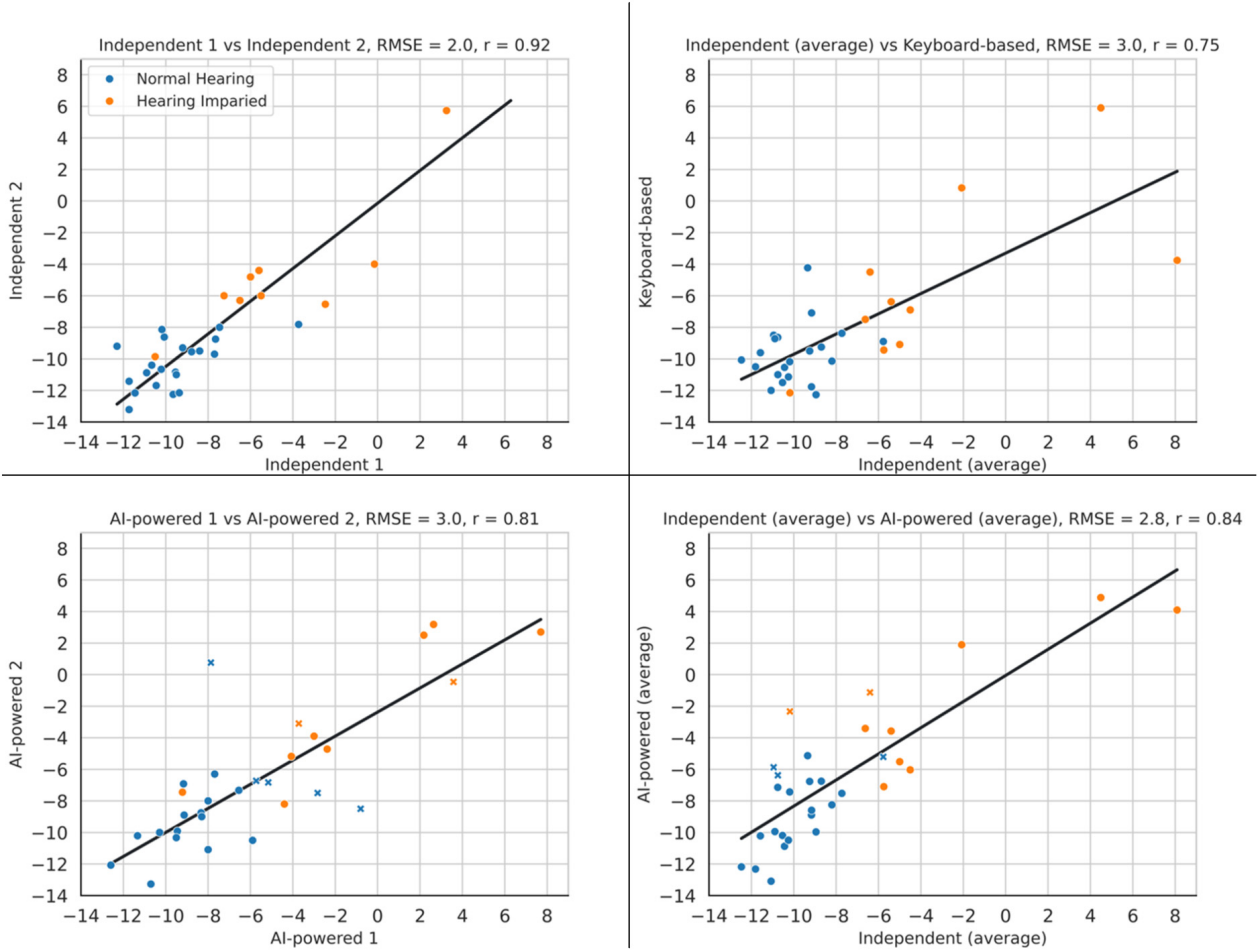
Comparison of the Correlation Plot of the Different Methods of the DIN Test. in This Plot, the Blue Marker Represent Participants with Normal-Hearing, the Orange Marker Represent Participants with Hearing Loss. the Markers Shown as Cross are the Outliers with High ASR Error Rate. In This Plot, RMSE Stands for Root-Mean-Square Error and r Represents Pearson's Correlation Coefficient.

The baseline for validity is defined by the differences between the two conventional tests, the independent and the keyboard-based tests, Figures 4 and 5 (top right). However, in this data, there was one participant with 11.8 dB difference between their results of the independent test and the keyboard-based test. To define a more representative baseline and avoid creating a baseline with unrealistically high LoA, the baseline was defined after removing this data point (LoA: −0.2 ± 4.4 dB, r: 0.82).

The baseline for reliability is the test–retest reliability of the Independent test. Figures 4 and 5 (top left) provide the results of this comparison, which are LoA of ±3.8 dB, mean of −0.4 dB and Pearson correlation of 0.92.

### Evaluating the Effect of Adding AI

To measure the effect of adding AI to the DIN test, validity (i.e., similarity of results to a target test) and test-retest reliability (i.e., consistency between two runs of the same test) of the AI-powered test were measured and compared to the baseline.

#### Validity

The effect of adding the ASR and TTS was measured by comparing the similarity of both the AI-powered and keyboard-based DIN with that of the Independent test using both correlation and B-A plots. As explained above, the top right plots in Figures 4 and 5 show the difference between the independent and the keyboard-based test before any AI was added to the system. The bottom right plot in those figures shows the difference between the independent and AI-powered tests, the two tests with the most differences in their methods.

After excluding the outlier data from the baseline, the AI-powered test showed slightly worse performance than the keyboard-based test in B-A analysis and RMSE (LoA increased from ±4.4 dB to ±4.9 dB; RMSE from 2.2 dB to 2.8 dB). However, it performed marginally better in terms of the Pearson correlation coefficient (increasing from 0.82 to 0.84). Overall, the results across all metrics were comparable, indicating that the inclusion of AI did not negatively affect performance.

**Figure 6.**
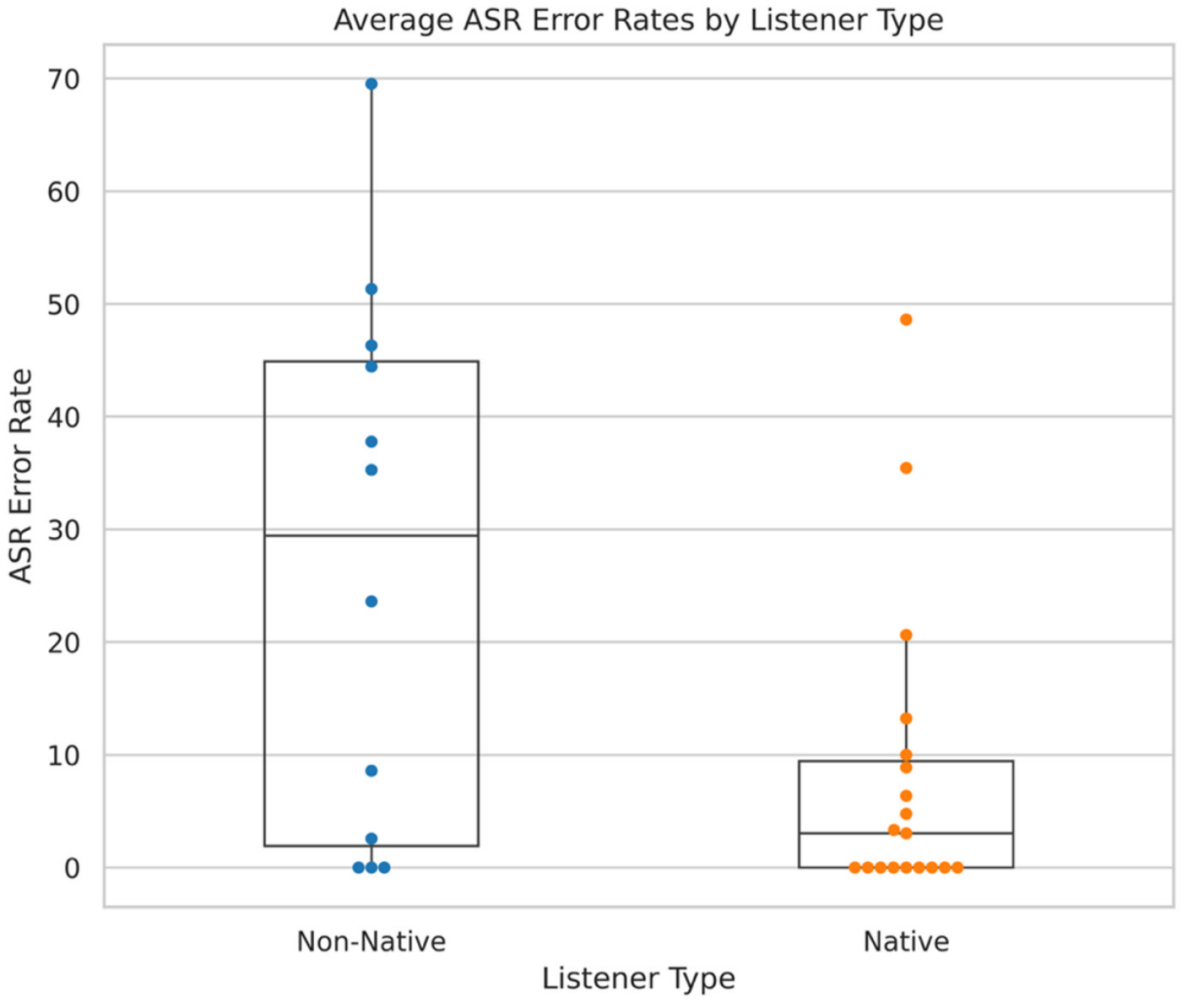
Showing the Average ASR Error of all the Participants Across the Three Runs of the AI-Powered Test.

Figure 6 shows the average error rates for all participants, along with the median and interquartile range. Five participants (16%) with error rates of 44%, 51%, 46%, 48% and 69% were excluded. This filtering improved the results for both B-A and correlation, with LoA improving from ±4.9 dB to ±3.9 dB, the Pearson correlation coefficient increasing from 0.84 to 0.91, and RMSE decreasing from 2.8 dB to 2.0 dB. Thus, more reliable test performance can be expected when it is known that the ASR performs well for a given participant.

#### Reliability

The reliability of the AI-powered test is compared with the reliability of the Independent test. The bottom left panel in Figures 4 and 5 show the similarity of results of the first and second runs of the AI-powered test. Plots show that the AI-powered test has a test-retest reliability worse than that of the Independent test (LoA: from ±3.8 dB to ±5.7 dB, Pearson correlation coefficient: from 0.92 to 0.81, RMSE: from 2.0 dB to 3.0 dB).

However, excluding participants with a high ASR error rate significantly improved the results. Filtering the participants results in excluding seven participants (22%), whose error rates were 44%, 55%, 80%, 81%, 83%, 83% and 100%. These error rates differ from those shown in Figure 6, as they reflect the highest error from either the first or second run, rather than the average error across all three runs. Excluding these participants results in the both having the same LoA of ±3.8 dB, but the AI-powered test has a somewhat higher mean difference (−0.9 dB vs −0.4 dB). In the correlation plot, their correlation coefficients are again the same (0.92) and the RMSEs are also very close (2.0 dB vs 2.1 dB). This shows that the AI-powered test is as consistent as the Independent test, as long as the ASR is performing well.

### TTS and ASR Evaluation

#### TTS Evaluation

**Figure 7.**
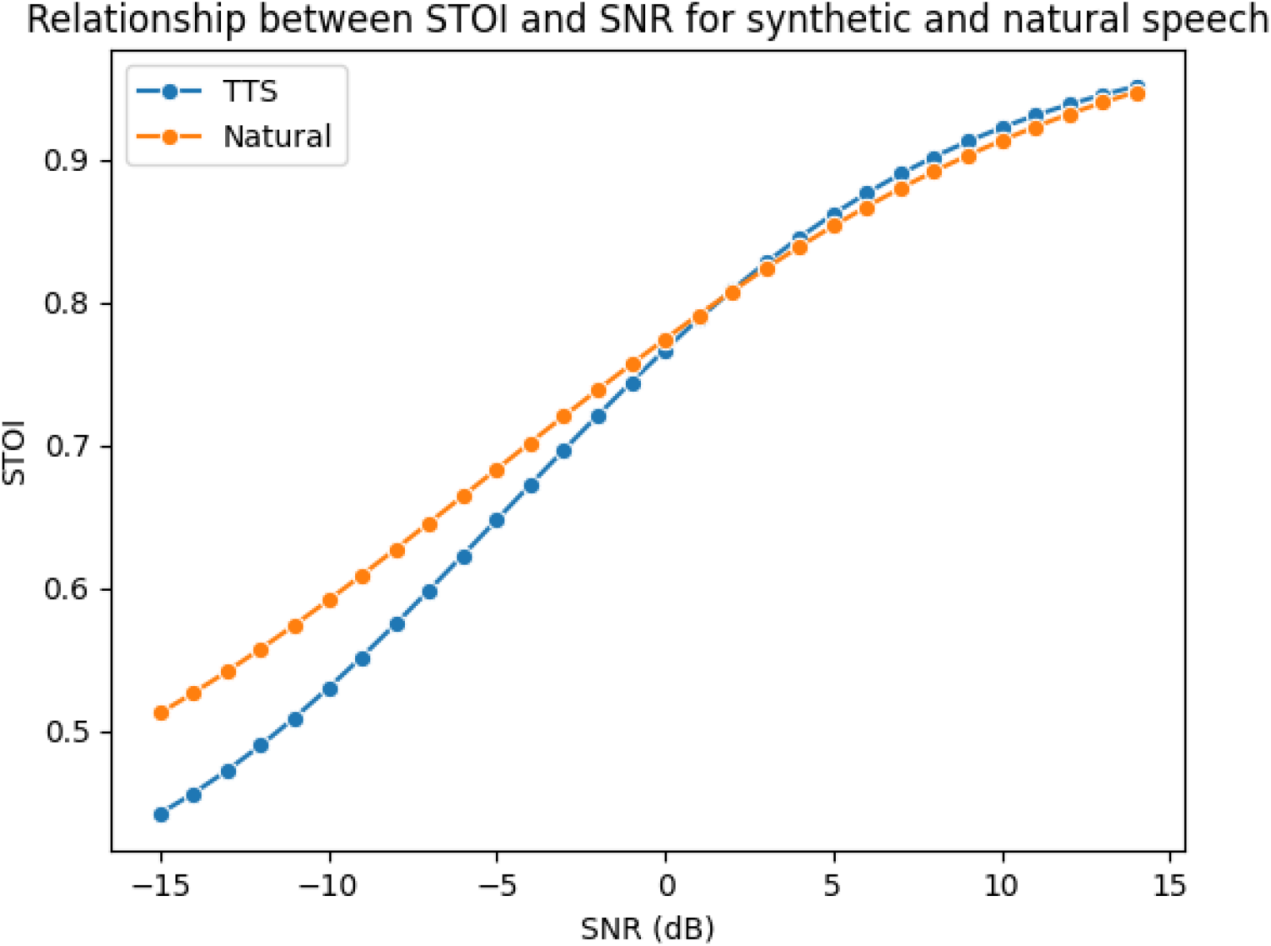
Average STOI Score of 30 Randomly Selected Stimuli for SNR Ranging from −10 to 10 with a Step Size of 1 dB.

Figure 7 presents the average STOI scores for randomly selected natural (from the Independent test) and synthetic stimuli. As expected, STOI decreased at lower SNRs and increased at higher SNRs. More importantly, the results showed that synthetic stimuli achieved STOI scores comparable to natural recordings.

NISQA-TTS was also used to assess the naturalness of the generated stimuli. On average, the natural stimuli received a MOS score of 3.5 ± 0.15, while the synthetic stimuli had a MOS of 3.5 ± 0.17.

These two metrics demonstrated that the synthetic stimuli closely matched the performance of the natural stimuli, indicating that the synthetic speech generation method preserved speech intelligibility at varying SNRs and was able to maintain a high level of naturalness and quality.

#### ASR Evaluation

By transcribing participants’ responses with the Whisper model, the ASR error was significantly reduced. The average ASR error rate dropped from 14.9% to 1.5%, with only five participants experiencing errors due to ASR mistakes at all, the highest being 17.6%. In contrast, 19 participants had ASR errors when using the simpler ASR model, with the highest error rate reaching 69.5%.

As mentioned earlier, ASR error rate is calculated using Equation (1), which yields a higher result compared to the conventional word error rate. For comparison, a total of 9396 single digits were presented to the participants and only 121 digits were transcribed incorrectly (1.2%) by the ASR. A complete list of ASR errors is available as supplementary material.

## Discussion

This proof-of-concept study proposed and validated a self-supervised English-language DIN with synthesized speech stimuli and ASR. Using synthetic stimuli means that stimuli can be easily produced as long as a high-quality TTS model is available. Using ASR instead of the usual numeric keypad to capture participants’ responses makes the test more accessible for people with certain disabilities (e.g., vision or mobility impairments) and those uncomfortable with computers and/or their interfaces.

The tests here provide evidence that other SIN tests with complex stimuli in which ASR would be the most intuitive method of registering participants’ responses (e.g., sentences), may be possible. Furthermore, our program has been made open-source and is freely available to customise and use by other researchers, which can facilitate research on this topic towards more complex SIN tests.

### Validity and Reliability

To evaluate the impact of adding ASR and TTS, we compared the results of the AI-powered test with a previously defined baseline test. Although the LoA for the baseline validity and reliability appears high, they align with findings reported by other researchers (Vroegop et al., 2021), reflecting the inherent variability of the DIN test. Moreover, since the DIN test is primarily used as a screening tool (De Sousa et al., 2020, 2022), it is still useful for that purpose despite the relatively large LoA.

Regarding the test validity, the addition of AI did not significantly affect the results, even with outliers included, and performance further improved once outliers were removed. Additionally, results from the Whisper model showed that removing participants with more than 40% was not an unrealistic condition and that further improvements are achievable with newer, state-of-the-art ASR models.

The AI-powered test was less reliable than the Independent test, but its reliability becomes almost identical once the outliers are removed. This shows that, if the ASR performs well, the AI-powered test is as reliable as the Independent test. One thing to consider is that the error-rate threshold for deleting a participant was set at 40%, which means that the system does not depend on perfect conditions to work and can handle some level of mistakes made by the ASR system.

A direct comparison of our model with other studies is not possible due to different methods of evaluation and pools of human participants. However, examining what other studies have achieved can still be useful for evaluating the results. Table 4 provides a summary of studies that used ASR for capturing participants’ responses.

**Table 4.** Methods That Used ASR for Capturing Participants’ Responses.

Study	Stimuli	Listeners	Stimuli Generation	Validity	Reliability
Ooster et al. (2018)	German words (Wagener et al., 1999)	27 native German speaking	Human recording	NH: 0.5 dB HI: 0.8 dB	NH: 0.5 dB HI: 0.9 dB
Bruns et al. (2022)	German words (Wagener et al., 1999)	16 native German speaking	Human recording	R = 0.93 NH: 1 dB	Unknown
Ooster et al. (2023)	German words (Wagener et al., 1999)	73 native German speaking	Human recording	Below 1.38 dB for 95% of the users	Unknown
Araiza-Illan et al. (2024)	Dutch digits triplet	6 native Dutch speaking	Human recording	Estimated <0.7 dB if ASR make less than 4 mistakes	Unknown
Ooster et al. (2020)	German words (Wagener et al., 1999)	46 native German speaking	Synthetic	B-A: Bias = 1.4 dB, LoA: ± 2.63 dB	NH: 0.63 dB HI: 1.01dB
**This study**	**English digits triplet**	**31 participants with different first language**	**Synthetic**	**R** **=** **0.91; B-A: bias** **=** **0.6** **dB, LoA:** **±** **3.9 dB**	**R** **=** **0.92; B-A: bias** **=** **−0.9** **dB, LoA:** **±** **3.8 dB**

R: Pearson correlation coefficient, NH: Normal-hearing, HI: Hearing-impaired. B-A: Bland-Altman, LoA: Limits of agreements.

The closest experiment to the current study was done by Araiza-Illan et al. (2024) in which they used a Dutch-language digit triplet. They assessed the ASR performance using responses from 30 native Dutch speakers, then selected the ASR-controlled DIN test results from six participants with no ASR errors and used bootstrapping to simulate the DIN test. They reported that if the ASR system makes up to four errors, the DIN test would produce clinically valid results.

Although comparing the results of different studies is not entirely accurate due to different measurement and evaluation methods, comparing our test with the Independent test and other studies shows that our developed software is working as expected and can reliably be used for performing DIN tests. Additionally, it showed that both TTS and ASR, despite having some shortcomings, are suitable for performing self-supervised DIN tests that can be easily conducted by participants themselves.

### Limitations

This proof-of-concept study had limitations in both the design and use of AI tools. One of the main points of implementing a self-supervised hearing test is to enable people to perform at-home assessment of their hearing. However, our testing was done in a sound-treated room and with calibrated equipment. These types of equipment are not available in participants’ homes (Peng et al., 2022), and for the intended final use case of self-administered hearing tests, more research is required under normal living conditions. Additionally, we used digits as stimuli. The limited vocabulary simplified the task for TTS by lowering the complexity of the speech to be synthesised. It also benefited ASR by narrowing the range of possible word choices and reducing sensitivity to accent variation. However, the lack of contextual information may have made recognition more difficult for the ASR system. With advances in those two technologies, they should be capable of accurately synthesising, and recognising, normal conversation. Therefore, we believe that they can be used with more complex stimuli (e.g., words and sentences).

It is common in studies that used TTS to check the quality of synthesised stimuli by humans (Ibelings et al., 2022; Nuesse et al., 2019; Ooster et al., 2020; Polspoel et al., 2025). However, we aimed to evaluate how TTS would perform with minimum human interference. Therefore, we chose not to check the quality of the generated stimuli before presenting them to the participants. A TTS system capable of consistently producing high-quality stimuli without the need for manual checking by humans would be particularly useful for future self-supervised conversational hearing tests (similar to a chatbot that can have a conversation with the participant), in which it is not possible to create pre-generated and verified stimuli, and the generated stimuli must be created based on the participant's response. However, not checking synthesised stimuli could have resulted in some synthesised numbers being distorted and incomprehensible, leading to incorrect responses that were not the participant's fault. However, steps, such as using ASR to evaluate the intelligibility of the synthesised speech or using automatic and non-intrusive systems to estimate TTS performance (Hermansky et al., 2013) can be taken to ensure its quality.

Using ASR also has some challenges, particularly in handling different accents from respondents. This caused problems in this study too, despite the limited vocabulary. Solving this problem is complex and remains an active area of research (Dhanjal & Singh, 2023).

While the Whisper model—trained on a large and diverse dataset that includes non-native speakers—showed that modern ASR systems can significantly reduce recognition errors, there is still room for improvement. One potential enhancement is the use of multimodal ASR systems that combine audio and visual inputs for transcription. Another is introducing a brief training phase for each user, allowing the system to adapt to individual voice characteristics and accents.

A procedure for evaluating the accuracy of the ASR at the start of the session can also be included. This could involve having the participant repeat various three-digit numbers displayed on the screen. Their response could then be used for evaluating the ASR and to check if the AI-powered test can be used for this person or not.

Limiting the participants to people with a PTA less than 55 dB HL can also cause problems for a fully self-supervised and automated test. One solution to solve this can be to implement a dynamic stimulus and noise level and let the participants adjust the level so that they can hear and understand the stimuli at the start of the test.

The mentioned limitations and shortcomings were the result of limited time, resources, and limitations of state-of-the-art ASR models. However, we will consider the mentioned limitations in designing our future studies to improve the designed system.

## Conclusions

In this proof-of-concept study, we developed a software application capable of conducting DIN tests using a combination of TTS and ASR and assessed the impact of these technologies on the SRT measurement. By comparing our proposed test with an Independent test we showed that our test is both reliable and valid for the performing of a DIN test. Future developments need to increase the accuracy of the TTS and ASR systems and explore options for creating more natural SIN tests.

## Supplemental Material

sj-json-1-tia-10.1177_23312165251367625 - Supplemental material for Digits-In-Noise Hearing Test Using Text-to-Speech and Automatic Speech Recognition: Proof-of-Concept Study Supplemental material, sj-json-1-tia-10.1177_23312165251367625 for Digits-In-Noise Hearing Test Using Text-to-Speech and Automatic Speech Recognition: Proof-of-Concept Study by Mohsen Fatehifar, Kevin J. Munro, Michael A. Stone, David Wong, Tim Cootes and Josef Schlittenlacher in Trends in Hearing

sj-csv-2-tia-10.1177_23312165251367625 - Supplemental material for Digits-In-Noise Hearing Test Using Text-to-Speech and Automatic Speech Recognition: Proof-of-Concept Study Supplemental material, sj-csv-2-tia-10.1177_23312165251367625 for Digits-In-Noise Hearing Test Using Text-to-Speech and Automatic Speech Recognition: Proof-of-Concept Study by Mohsen Fatehifar, Kevin J. Munro, Michael A. Stone, David Wong, Tim Cootes and Josef Schlittenlacher in Trends in Hearing

sj-zip-3-tia-10.1177_23312165251367625 - Supplemental material for Digits-In-Noise Hearing Test Using Text-to-Speech and Automatic Speech Recognition: Proof-of-Concept Study Supplemental material, sj-zip-3-tia-10.1177_23312165251367625 for Digits-In-Noise Hearing Test Using Text-to-Speech and Automatic Speech Recognition: Proof-of-Concept Study by Mohsen Fatehifar, Kevin J. Munro, Michael A. Stone, David Wong, Tim Cootes and Josef Schlittenlacher in Trends in Hearing

## Notes

1.
https://huggingface.co/speechbrain/asr-crdnn-rnnlm-librispeech

2.
https://huggingface.co/speechbrain/tts-hifigan-ljspeech
